# Whole-genome sequencing of Listeria monocytogenes from maternal and neonatal clinical isolates in Kuwait

**DOI:** 10.1099/jmm.0.002181

**Published:** 2026-06-29

**Authors:** Ola H. Moghnia, Aisha M. Al-Haqqan, Hessah S. Al-Otaibi, Abdallah B. El-Kurdi, Habiba Y. Mohammed, Ali Karkaba, Haneen Y. Mohammed, Noura A. Al-Sweih

**Affiliations:** 1 Department of Microbiology, College of Medicine, Kuwait University, Kuwait City, Kuwait; 2 Microbiology Laboratory, Maternity Hospital, Ministry of Health, Kuwait City, Kuwait; 3 Department of Biochemistry and Molecular Genetics, American University of Beirut, Beirut, Lebanon; 4 College of Medicine, Kuwait University, Kuwait City, Kuwait; 5 Faculty of Biotechnology, School of Arts and Sciences, American International University, Kuwait City, Kuwait; 6 Cognosco, Anexa Veterinary Services, Morrinsville, New Zealand

**Keywords:** *Listeria monocytogenes*, whole-genome sequencing, antimicrobial resistance, maternal listeriosis, neonatal listeriosis, Kuwait

## Abstract

**Introduction.** *Listeria monocytogenes* is a foodborne pathogen that poses a significant threat during pregnancy, frequently associated with adverse maternal and neonatal outcomes, including preterm birth, spontaneous abortion, stillbirth and neonatal sepsis.

**Hypothesis/Gap Statement.** Despite its clinical relevance, there is a lack of whole-genome sequencing (WGS)-based data describing the genomic characteristics and circulating lineages of *L. monocytogenes* associated with maternal and neonatal infections in Kuwait, limiting regional epidemiological understanding.

**Aim.** To genomically characterize *L. monocytogenes* isolates from maternal and neonatal clinical specimens at the Maternity Hospital, Kuwait.

**Methodology.** Nine clinical isolates collected between 2017 and 2022, obtained from high vaginal swabs and blood cultures of unrelated mothers and neonates, were subjected to WGS. Antimicrobial susceptibility testing was performed using the VITEK-2 system. Genomic analysis included determination of sequence types (STs), clonal complexes (CCs), genomic characterization using multi-locus sequence typing (MLST), core-genome MLST (cgMLST), antimicrobial resistance (AMR) determinants and virulence gene profiles.

**Results.** Seven isolates (S2–S8) were ST2 and belonged to CC2, whereas S1 and S9 were classified as ST3 and ST308 and belonged to CC3 and CC1, respectively. Phenotypic susceptibility profiles were correlated with genotypic findings, with a conserved resistome dominated by *fosX* (fosfomycin resistance) and *vga(G*) (lincosamide resistance). All isolates harboured conserved core virulence genes associated with stress response, quorum sensing, nutrient regulation, host cell invasion and intercellular survival. The cgMLST analysis demonstrated ≥99.5% genetic similarity among ST2/CC2 isolates, which clustered together and were genetically distinct from older local isolates (S1, S9) and international reference strains.

**Conclusion.** This first WGS-based genomic characterization of *L. monocytogenes* in Kuwait demonstrates the repeated detection of ST2/CC2 across multiple maternal and neonatal cases over several years. These findings provide baseline genomic data and highlight the need for integrated, expanded genomic surveillance linking clinical, food and environmental isolates to improve source attribution, early detection and public health interventions for listeriosis.

**Impact Statement**

This study presents the first whole-genome sequencing-based characterization of *Listeria monocytogenes* isolates from maternal and neonatal clinical cases in Kuwait. The identification of three sequence types, with a predominance of ST2/CC2, is indicative of repeated circulation of *L. monocytogenes* over time.

## Data Summary

All data relevant to this study are available within the manuscript and its supplementary materials. Raw sequencing data have been deposited in the National Center for Biotechnology Information Sequence Read Archive (SRA) and are publicly available at https://www.ncbi.nlm.nih.gov/sra/ under the BioProjects accession number PRJNA1021349. The corresponding SRA accession numbers for each isolate are as follows: S1: SRX21900731; S2: SRX21900730; S3: SRX21900729; S4: SRX21900728; S5: SRX21900727; S6: SRX21900726; S7: SRX21900725; S8: SRX21900724 and S9: SRX21900723.

Additional data supporting the findings of this study are not publicly available due to the inclusion of information that could compromise the privacy of research participants. However, these data are available from the corresponding author upon reasonable request.

## Introduction

*Listeria monocytogenes* is a facultative intracellular, Gram-positive foodborne pathogen associated with severe invasive disease and high hospitalization and mortality rates, mainly among susceptible populations such as immunocompromised individuals, pregnant women and neonates [1]. Pregnancy is a well-recognized risk factor for listeriosis, with reported incidence rates 10–100-fold higher than in the general population and an estimated 18-fold increased risk during gestation, reflecting pregnancy-associated alterations in cell-mediated immunity [2,3].

Although maternal and neonatal listeriosis are frequently diagnosed in hospital settings, infections typically originate in the community through foodborne exposure. Pregnant women may acquire *L. monocytogenes* via contaminated food, leading to gastrointestinal colonization followed by haematogenous spread. Once in the bloodstream, the pathogen can cross the placental barrier, resulting in adverse outcomes including spontaneous miscarriage, stillbirth, preterm labour, neonatal sepsis and meningitis [4]. The incidence of neonatal listeriosis is estimated at ~8.6 per 100,000 live births, with reported mortality rates ranging from 20 to 60%, making it one of the leading causes of neonatal bacterial meningitis [5]. Thus, maternal infection represents a critical interface between community-acquired exposure and the hospital-based clinical presentation, in which disease manifests as obstetric or neonatal complications rather than through in-hospital transmission. This dual pathway highlights the importance of distinguishing foodborne acquisition from healthcare-associated spread when evaluating maternal and neonatal listeriosis cases [6]. Despite its clinical severity, maternal and neonatal listeriosis remains underreported globally, largely due to nonspecific maternal symptoms, diagnostic challenges and limited systematic surveillance.

In recent years, whole-genome sequencing (WGS) has emerged as a powerful tool for listeriosis surveillance, enabling high-resolution strain typing, outbreak detection and characterization of antimicrobial resistance (AMR) and virulence determinants [7]. However, WGS-based studies of *L. monocytogenes* remain limited in many regions, including Kuwait, limiting insights into local genomic diversity, circulating lineages and epidemiological patterns.

In this study, we characterized the genomic features of *L. monocytogenes* isolates recovered from maternal and neonatal clinical cases in a major maternity hospital in Kuwait. Using WGS-based approaches, we examined phylogenetic relationships, AMR profiles and virulence determinants. By providing the first genomically resolved data from Kuwait, this study contributes to the understanding of maternal and neonatal listeriosis and highlights the value of genomic surveillance for informed public health preparedness and clinical management.

## Methods

### Study design, sample collection and study population

Between 2017 and 2022, a total of nine clinical *L. monocytogenes* isolates were collected from seven mothers and two neonates admitted to Maternity Hospital, Ministry of Health, Kuwait. The Maternity Hospital is a major tertiary care centre, accounting for around one-third of deliveries in Kuwait and providing comprehensive obstetric, gynaecologic and neonatal care. Isolates were obtained from high vaginal swabs (HVS; *n*=2) and blood cultures (*n*=7) as part of the routine diagnostic evaluations for suspected sepsis cases. Samples originated from multiple hospital units, including labour rooms, general wards, intensive care units (ICUs) and neonatal intensive care units (NICUs). All isolates were transported to the Department of Microbiology, College of Medicine, Kuwait University, for further laboratory and genomic analyses. Prior to analysis, all samples were fully anonymized.

### Bacterial isolation and identification

Clinical specimens were processed using standard microbiological techniques [8]. Positive blood culture bottles were initially examined by Gram staining and subsequently analysed using automated molecular diagnostic platforms, including the BioFire^®^ FilmArray^®^ system (BioFire Diagnostics, USA), a multiplex PCR-based assay or the Verigene^®^ system (Nanosphere Inc., USA), which uses microarray-based technology for pathogen detection and resistance gene profiling. Both systems were operated according to the manufacturer’s protocols.

In parallel, samples were subcultured onto blood agar plates and incubated under appropriate conditions to promote the growth of *Listeria* species. HVSs were similarly subcultured onto blood agar and incubated at 35–37 °C for 24–48 h under aerobic conditions [8]. Species-level identification of all isolates was confirmed using the VITEK-2 automated system (bioMérieux, Marcy-l’Étoile, France) with ID-GPC cards for Gram-positive bacteria, according to the manufacturer’s recommendations.

### Antimicrobial susceptibility testing

Antimicrobial susceptibility testing was assessed using the VITEK-2 automated system (bioMérieux, Marcy-l'Étoile, France). The antimicrobial panel included ampicillin, penicillin-G, oxacillin, gentamicin, ciprofloxacin, teicoplanin, vancomycin, fusidic acid, sulfamethoxazole-trimethoprim, piperacillin-tazobactam, erythromycin, clindamycin, cloxacillin and nitrofurantoin. Results were interpreted using VITEK-2 software (version VTK-R01.02) in accordance with European Committee on Antimicrobial Susceptibility Testing (EUCAST) clinical break points version 12 [8].

### Whole-genome sequencing and genomic analysis

Genomic DNA was extracted using the QIAamp DNA Mini Kit (QIAGEN, Valencia, USA), according to the manufacturer’s instructions, edition-Track as previously described [9]. Genomic DNA concentration and purity were assessed using a NanoDrop-800 spectrophotometer (Thermo Fisher Scientific, Wilmington, DC, USA), and DNA integrity was confirmed by 0.8% agarose gel electrophoresis.

Sequencing libraries were prepared using the Nextera DNA Sample Preparation Kit (Illumina, San Diego, CA, USA). Fragment sizes were selected in the 200–400 bp range, followed by amplification (12 PCR cycles) and purification using AMPure XP beads (Beckman Coulter, Indianapolis, USA). Library quality was evaluated using a Qubit fluorometer (Thermo Fisher Scientific) and an Agilent 2100 Bioanalyzer for fragment size analysis. Paired-end sequencing (2×150 bp) was performed on the BGISEQ-500 platform. Raw sequencing reads were evaluated for quality using FastQC [10], and *de novo* genome assembly was performed using SPAdes [11] with default parameters.

*In silico* analysis genomic characterization included multi-locus sequence typing (MLST) using the BIGSdb-Pasteur *Listeria* database (https://bigsdb.pasteur.fr/listeria/) [12,13]. Core-genome MLST (cgMLST) analysis was conducted using the chewBBACA pipeline [14]. The *L. monocytogenes* scheme was retrieved from https://chewbbaca.online/stats, and the allele calling was performed using the chewBBACA Allele Call module. Core loci were determined using the Extract cgMLST module, and a custom R script was used to generate a minimum spanning tree for the nine isolates.

Whole-genome MLST (wgMLST) phylogenetic analysis was conducted using the Cano-wgMLST pipeline [15]. Briefly, each isolate is annotated using PROKKA, and then a pan-genome is constructed using Roary, followed by allele typing and allelic profiling. The output of this pipeline is a phylogenetic tree in Newick format that was used to generate the phylogenetic tree using a custom R script. The publicly available datasets were downloaded from the following project IDs: PRJNA514245, which includes clinical and environmental isolates and PRJNA606479, which includes isolates from poultry. The corresponding profile IDs and metadata are provided in Table S4, available in the online Supplementary Material.

Assembled contigs were screened for AMR genes using AMRFinderPlus [16] and the Comprehensive Antibiotic Resistance Database Resistance Gene Identifier tool [17]. Virulence factors were identified using the Virulence Factors Database [18], and plasmid replicons were detected using PlasmidFinder [19]. Phylogenetic trees were visualized using the ggtree package in R/Bioconduction [20].

## Results

A total of nine clinical *L. monocytogenes* isolates were recovered from seven mothers and two neonates admitted to the Maternity Hospital in Kuwait between 2017 and 2022. Specimens comprised five maternal blood cultures (S1, S3, S4, S6 and S9), two HVSs (S5 and S7) and two neonatal blood cultures (S2 and S8). The isolates originated from multiple hospital units, including the NICUs (S2, S8), ICU (S4), labour rooms (S1, S3, S5, S6) and ward 19 (S7, S9). Temporally, isolate S1 was collected in 2017, S9 in 2018, S2 and S4 in 2021 and the remaining five isolates in 2022 (Table 1).

**Table 1.** Antimicrobial resistance patterns and metadata of *L. monocytogenes* isolates from maternity hospital in Kuwait (2017–2022)

Sample	Collection date	Location	Specimen type	Antimicrobial susceptibility profile
**S1**	6 September 2017	LR2	B/C	Clindamycin resistant only
**S2**	29 November 2021	NICU1	B/C	All susceptible
**S3**	18 June 2022	LR2	B/C	All susceptible
**S4**	17 October 2021	ICU	B/C	All susceptible
**S5**	2 November 2022	LR1	HVS	All susceptible
**S6**	19 June 2022	LR2	B/C	All susceptible
**S7**	13 December 2022	Ward 19	HVS	All susceptible
**S8**	3 May 2022	NICU2	B/C	Clindamycin resistant only
**S9**	30 June 2018	Ward 19	B/C	All susceptible

Antimicrobials tested were ampicillin, penicillin-G, oxacillin, gentamicin, ciprofloxacin, teicoplanin, vancomycin, fusidic acid, sulfamethoxazole-trimethoprim, piperacillin-tazobactam, erythromycin, clindamycin, cloxacillin and nitrofurantoin.

B/C, blood culture; LR, labour Room.

Antimicrobial susceptibility testing showed that all isolates were susceptible to first-line agents, including ampicillin and penicillin G. Resistance was observed in two isolates, S1 (2017, labour room) and S8 (2022, Neonatal ICU), both of which were resistant to clindamycin (Table 1).

### Genome assembly and annotation

WGS of *L. monocytogenes* isolates revealed sizes ranging from 2.75 to 2.82 Mbp and a G+C content of 38.08–38.14 mol%. All isolates contained 3 rRNA operons, 53 tRNA genes and a single transfer messenger RNA, except isolate S1, which harboured 8 rRNA operons and 94 tRNA genes. Detailed genome assembly and annotation statistics are provided in Table 2.

**Table 2.** Genomic characteristics of assembled and annotated *L. monocytogenes* draft genomes

S9	S8	S7	S6	S5	S4	S3	S2	S1	Feature
**Assembly statistics**									
69	76	79	77	76	74	78	79	88	Total contigs (≥500 bp)
49	64	65	63	61	60	62	65	69	Contigs>1 kb
2,751,849	2,759,139	2,777,768	2,768,390	2,770,847	2,774,153	2,798,311	2,782,729	2,817,262	Total assembly length (bp)
38.12	38.14	38.12	38.14	38.14	38.13	38.11	38.12	38.08	G+C content (mol%)
99,561	97,377	99,529	97,309	97,734	99,529	99,529	97,903	150,638	N50 (bp)
**Annotation statistics**									
2,682	2,704	2,723	2,709	2,718	2,715	2,749	2,731	2,713	CDS
8	3	3	3	3	3	3	3	3	rRNA
94	53	53	53	53	53	53	53	53	tRNA
1	1	1	1	1	1	1	1	1	tmRNA

CDS, coding sequence; tmRNA, transfer messenger RNA.

### Multi-locus sequence typing (MLST)

MLST analysis revealed three sequence types (STs) among the nine *L. monocytogenes* isolates, all belonging to Lineage I. Seven isolates collected between 2021 and 2022 (S2–S8, 77.8%) were assigned to ST2 within clonal complex 2 (CC2). Isolate S1, collected in 2017, was classified as ST3 (CC3); instead, isolate S9, collected in 2018, belonged to ST308 (CC1). Detailed allelic profiles and ST assignments are provided in Table S1.

### Resistance and virulence factor profiling

Resistance gene analysis revealed a limited profile, with all isolates harbouring *fosX* (fosfomycin resistance) and *vga(G*) (lincosamide resistance). All isolates shared a highly conserved core virulence repertoire, including the master regulator *prfA*, stress response regulators (*sigB* and *virR*), quorum sensing (*agrA*) and the global metabolic regulator *codY*. Genes encoding internalins (*inlA, inlB, inlC* and *inlJ*), pore-forming toxins and phospholipases (*hly, plcA, plcB* and *mpl*), stress adaptation (*gadB*, *gadC*, *clpB*, *clpC*, *clpE*, *clpP*, *htrA* and *bsh*) and iron/nutrient acquisition systems (*fur*, *hupC*, *oppA*) were also conserved across all isolates.

Notably, isolate S1 exhibited an expanded virulence profile, including *actA*, along with additional genes such as *aut, ami*, *gadA* and *gtcA*, which were absent from the remaining isolates. In contrast, isolates S2–S8 shared identical core virulence profiles, whereas S9 lacked *actA*. A complete virulence gene summary is provided in Table S2.

A set of conserved housekeeping and fitness-associated genes is present in all isolates, including those involved in DNA repair (*recA*), cell wall synthesis (*murA, lsp* and *lgt*) and protein secretion and folding (*secA2, prsA2, tig* and *sipX/Z*). Genes supporting central metabolism (*pycA, lplA* and *pgl*) and general stress responses (*sod* and *per*) were also universally detected. In addition, all isolates carried fbpA, encoding a fibronectin-binding protein involved in host cell adhesion, and *hfq*, an RNA chaperone implicated in post-transcriptional regulation and stress adaptation.

### Core-genome MLST (cgMLST)

cgMLST analysis showed that isolates S2–S8 formed a highly related cluster, all sharing the same cgMLST profile (cgMLST profile ID: 21857- generated internally by the BIGSdb pipeline). These isolates exhibited 1,728–1,741 allele matches across 1,748 loci (99.5–99.7% similarity) from the Institute Pasteur BIGSdb database (https://bigsdb.pasteur.fr/cgi-bin/bigsdb/bigsdb.pl?db=pubmlst_listeria_seqdef&page=sequenceQuery, where each isolate is compared to the reference allele scheme, indicating they are genetically closely linked). In contrast, isolate S1 (profile ID: 20537) had 1,728 allele matches (97.9% similarity) and was distinct from the clusters. Isolate S9 (profile ID: 20879) had 1,741 allele matches (99.6% similarity) with a different profile (Table S3).

### Whole-genome multi-locus sequence typing (wgMLST) and cgMLST minimum spanning tree

**Fig. 1.**
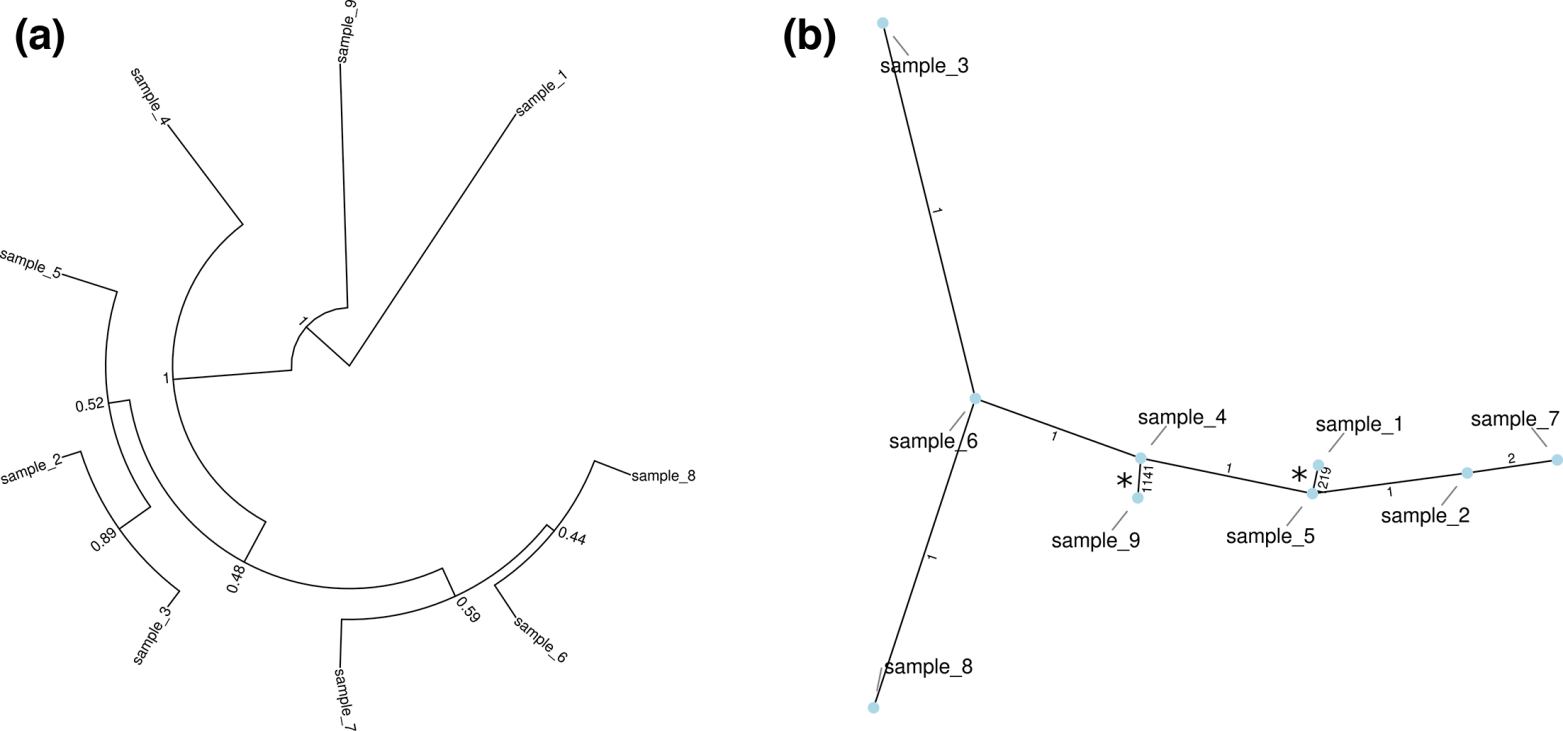
(a) wgMLST phylogenetic tree generated using the Cano-wgMLST pipeline with bootstrapping values added to the branches. (b) cgMLST minimum spanning tree (MST) phylogenetic tree of the nine isolates using the output of the chewBBACA ‘extractCgMLST’ module after running the ‘AlleleCall’ module from the same package. It shows the evolutionary relationships between the isolates (nodes). The distance between the nodes represents evolutionary change. The numbers on the tree branches represent pairwise allelic distances between isolates. edition Cleanedit-Clean

Hierarchical clustering based on wgMLST revealed clear genetic relationships among the S2–S8 isolates, whereas S1 and S9 branched independently, indicating distinct genetic lineages (Fig. 1a). This clustering pattern corroborates the cgMLST results and is further supported by the cgMLST-based minimum spanning tree from the chewBBACA pipeline (AlleleCall followed by Extract cgmlst), which highlights the close relatedness of S2–S8 and the genetic separation of S1 and S9 (Fig. 1b). The numbers on the tree branches represent pairwise allelic distances between isolates based on the chewBBACA cgMLST core genome analysis, rather than similarity to the Institute Pasteur cgMLST reference scheme.

**Fig. 2.**
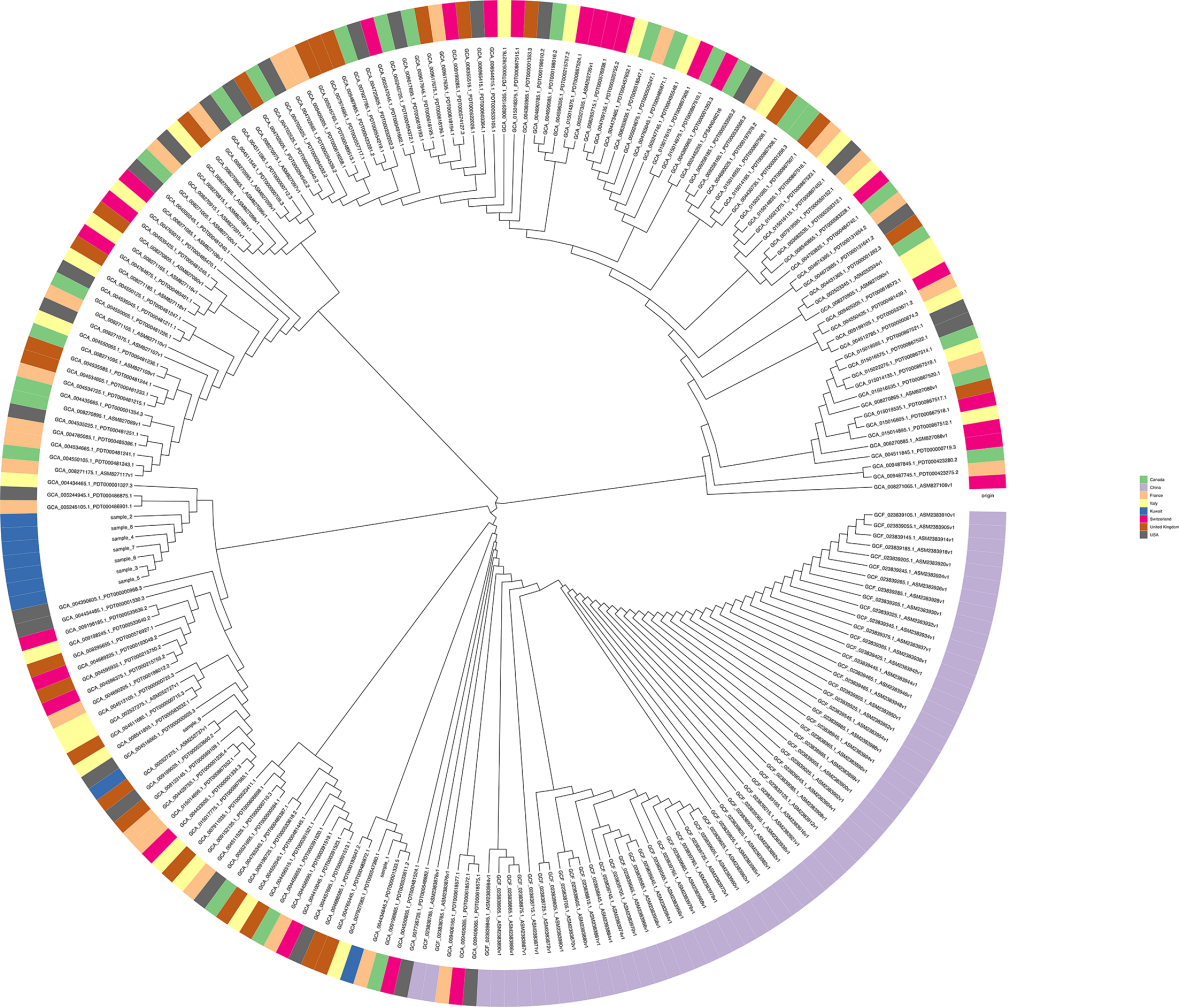
Phylogenetic and geographic analysis of global and local *L. monocytogenes* isolates. A maximum-likelihood phylogenetic tree was constructed based on a wgMLST profile, incorporating local isolates from this study (highlighted in blue) and a global collection of international reference strains retrieved from publicly available databases. Isolates are colour-coded by country of origin, as indicated in the legend.

To explore the geographic distribution between local isolates and globally circulating lineages of *L. monocytogenes* isolates. A comparative phylogenetic tree was generated using wgMLST allelic profiles from representative international reference strains encompassing both clinical isolates and environmental or food-associated isolates from publicly available repositories. Local isolates (highlighted in blue) were dispersed across the tree, clustering near strains from other countries such as France, the United States and Italy (Fig. 2). The global reference collection comprised strains originating from diverse sources, including human clinical cases, food products, food-processing environments and environmental samples (Table S4).

## Discussion

WGS of nine *L. monocytogenes* isolates obtained from maternal and neonatal cases in a tertiary maternity hospital in Kuwait revealed insights into genomic diversity, AMR profiles, virulence factors and phylogenetic relationships with regional and global strains. According to the World Health Organization, ~43% of reported *Listeriosis* cases occur during pregnancy, with 14% of those cases presenting in late gestation [21,22]. Pregnancy-associated hormonal changes, mainly elevated progesterone levels, suppress cell-mediated immunity, thereby increasing susceptibility to invasive *L. monocytogenes* infection [21]. Maternal and neonatal listeriosis is uncommon but poses a significant, severe outcome. Fetal infection occurs mainly through haematogenous spread following gastrointestinal colonization of the tract or, less frequently, through ascending genital tract infection, both of which may cause neonatal complications [23]. Maternal infection may be asymptomatic or may cause nonspecific symptoms such as malaise, fever, gastrointestinal disturbances or flu-like symptoms. However, fetal and neonatal manifestations can include sepsis, meningitis, preterm labour and miscarriage. Prompt detection of listeriosis and targeted antimicrobial therapy remain critical to improving clinical outcomes [24]. Despite its significance, systematic genomic surveillance of maternal and neonatal listeriosis remains largely absent and underreported in the region, where data are limited to sporadic case reports and scarce retrospective studies.

A 10-year retrospective study in Qatar reported that 22.9% of listeriosis cases were pregnancy-associated, with high fetal morbidity, including stillbirth (33%) and preterm delivery (55%), indicating that pregnancy is a significant risk factor for listeriosis [25]. Regional food surveillance studies report variable prevalence of *L. monocytogenes*, with contamination rates ranging from 1.6% in Iraq to 17.6% in Jordan, and raw milk contamination reaching up to 5.8%, indicating ongoing exposure risks [25]. Other reports from Egypt, Tunisia, Algeria and Saudi Arabia link *Listeria* carriage to adverse pregnancy outcomes, highlighting the pathogen’s impact on maternal health [26]. To our knowledge, this study represents one of the first WGS-based investigations of maternal and neonatal listeriosis in Kuwait, providing essential baseline genomic data.

Although the number of isolates analysed is limited, the temporal distribution (2017–2022) and recovery from multiple clinical units reflect sporadic case detection over several years rather than a confined outbreak. Owing to the retrospective study design, these cases involved unrelated mother and infant pairs; therefore, epidemiological linkage could not be established. In addition, the absence of food, environmental or veterinary isolates precluded definitive source attribution. Bloodstream isolates, although relatively uncommon for mothers, represent clinically significant maternal infection with potential fetal exposure, supporting their relevance for genomic characterization.

Genomic analyses revealed that isolates S2–S8, collected between 2021 and 2022, formed a tight genomic cluster despite originating from multiple hospital units. While the detection of genetically related isolates across time and location could reflect repeated exposure to a common source, such as contaminated food, no epidemiological data from food exposures, environmental or veterinary sources were available to confirm this hypothesis. Importantly, these findings do not support nosocomial transmission, but rather suggest multiple introductions of genetically similar strains, consistent with nationally or regionally circulating strains. In China, comparable findings have been reported, where ST8 isolates from maternal and neonatal sources clustered with foodborne strains from multiple regions [6].

Antimicrobial susceptibility testing demonstrated universal susceptibility to first-line agents, including ampicillin, gentamicin, penicillin G and vancomycin, supporting current neonatal and maternal listeriosis treatment guidelines [27]. Clindamycin resistance was detected in two isolates (S1 and S8), which both carried the *vga(G*) gene. While *vga*-mediated lincosamide resistance is well described in *Staphylococcus aureus* [28,29], discordance between genotype and phenotype in those isolates may reflect silent or low-level of *vga (G*) gene expression, regulatory effects or possible testing limitations, including VITEK-2 automated susceptibility testing errors [30,31]. Similar resistance patterns have been observed in other studies, underscoring the importance of phenotypic confirmation and cautious interpretation of automated AST results to guide targeted therapy and optimize clinical outcomes [6,32]. All isolates carried a conserved minimal resistome, including *fosX*, fosfomycin resistance and lacked plasmids, suggesting chromosomal integration or intrinsic resistance mechanisms. These findings align with previous WGS studies reporting limited acquired resistance determinants, such as *fosX*, *mprF*, *norB* and *sul,* but lacking plasmids in *L. monocytogenes* clinical isolates [6]. Another study reported that *L. monocytogenes* isolates harboured only intrinsic resistance genes, including *fosX*, *mprF* and *norB*, with no acquired AMR determinants detected [32].

Genome assembly metrics were consistent with reference *L. monocytogenes* genomes, including genome sizes of 2.75–2.81 Mb and G+C content of ~38.1 mol%. Isolate S1 exhibited a larger genome size and higher rRNA/tRNA copy numbers (8 rRNA operons, 94 tRNAs); this could be explained by a configuration previously reported in fully resolved genomes using long-read sequencing and reflective of repetitive region resolution rather than true genomic expansion [33]. G+C content was highly conserved across all isolates. Minor differences compared with earlier studies likely reflect strain-level and assembly-method variation [6].

Virulence profiling revealed that all isolates carried core pathogenic determinants (*prfA, hly, inlA/B, plcA/B*), essential for placental invasion and intracellular survival [34]. The conservation of those determinants reflects a robust metabolic and regulatory framework enabling *L. monocytogenes* persistence across diverse host environments. The presence of *gad* and *bsh* genes supports gastrointestinal persistence, consistent with reports of asymptomatic carriage of *L. monocytogenes* in ~4% of pregnant women [35,36].

In addition, the presence of *LLO* and *actA* genes mediates intracellular dissemination, phagosomal escape and cytosolic replication, resembling earlier reports of ST8 clinical isolates carrying *actA, prfA*, multiple internalins and stress-adaptation loci such as survival islet 1 (SSI-1) and *cadC* [37].

Isolate S1 displayed an expanded virulence repertoire, including *actA, aut, ami, gadA* and *gtcA*, suggesting strain-specific adaptations linked to pathogenicity or environmental persistence. In contrast, isolate S9 lacked *actA*, potentially limiting its intracellular spread. All isolates also carried *agrA,* implicated in biofilm formation and quorum sensing [29]. These findings align with prior reports linking core and accessory virulence factors in maternal and neonatal disease severity [6,37].

*L. monocytogenes* is a highly diverse bacterium, with its population structure categorized into 15 recognized serotypes and four phylogenetic lineages (I, II, III and IV), and these lineages were divided into CCs [32]. MLST analysis identified three CCs with ST2/CC2 predominance (78%) among isolates recovered in 2021–2022. In addition to ST3/CC3 (S1, 11%) and ST308/CC1 (S9, 11%).

Many studies reported that CC2/ST2 is prevalent and has been repeatedly associated with severe listeriosis [38,39]. Also, ST3 has been documented well in China [40]. Analysis of cgMLST confirmed that isolates S2–S8 formed a single highly related cluster (≥99.5% similarity), whereas the cgMLST profile and absence of key virulence expansions of earlier isolates (S1 and S9) suggest they were not part of the same transmission chain and represented genetically distinct introductions. Lineage I serotype 4b members (CC1, CC2, CC4 and CC6) are overrepresented in maternal and neonatal infections in France, accounting for more than two-thirds of cases [41].

Analyses of wgMLST and global phylogeny further demonstrated that the local isolates clustered with strains from France, Italy and the United States, suggesting shared international lineages rather than a unique endemic strain, although limited sampling and the absence of a national surveillance system for foodborne and environmental *Listeria* strains preclude firm conclusions about cross-border transmission. This study highlights the genomic heterogeneity of clusters of *L. monocytogenes* circulated among maternal and neonatal cases in Kuwait.

## Conclusions

This study provides the first baseline WGS data on *Listeria monocytogenes* associated with maternal and neonatal cases in Kuwait. The predominance of ST2/CC2 isolates detected across multiple cases and years indicates the circulation of genetically related strains; however, in the absence of food chain isolates, environmental or veterinary sampling, transmission pathways and sources cannot be inferred, emphasizing the need for ongoing integrated One Health surveillance. Expanded genomic monitoring of food, environmental and clinical isolates, coupled with strengthened antimicrobial stewardship and targeted public health messaging for pregnant women, is essential to reduce the burden of maternal and neonatal listeriosis and improve maternal and neonatal outcomes.

## Limitations

This study is limited by its relatively small sample size and the absence of parallel environmental, veterinary or food isolates, which restricts the ability to identify potential sources and transmission pathways of *Listeria monocytogenes*. In addition, the absence of a national WGS-based surveillance system for foodborne pathogens in Kuwait constrains outbreak detection, hinders cross-border comparisons and limits the integration of clinical and foodborne data needed for comprehensive public health surveillance.

## Supplementary material

10.1099/jmm.0.002181 Supplementary Material 1.

## Abbreviations

AMR: antimicrobial resistance
CC: clonal complex
CDS: coding sequence
cgMLST: core-genome multi-locus sequence typing
EUCAST: European Committee on Antimicrobial Susceptibility Testing
HVS: high vaginal swab
ICU: intensive care unit
LR: labour Room
MLST: multi-locus sequence typing
NICU: neonatal intensive care unit
SRA: sequence read archive
ST: sequence type
wgMLST: whole-genome multi-locus sequence typing
WGS: whole-genome sequencing
