# Supplementary material for: Whole-genome sequencing of Listeria monocytogenes from maternal and neonatal clinical isolates in Kuwait
Source: J Med Microbiol. 2026 Jun 29;75(6):002181. doi: 10.1099/jmm.0.002181 (PMC13313355; doi:10.1099/jmm.0.002181)
Supplement: Supplementary Material 1. [file jmm-75-02181-s001.pdf]

Supplementary materials

Table S1. Multi-locus Sequence Typing data of *Listeria monocytogenes* isolates.

**Sample**	**ST**	***abcZ***	***bglA***	***cat***	***dapE***	***dat***	***ldh***	***lhkA***	**CC**	**Lineage**
S2-S8	2	1	1	11	11	2	1	5	CC2	I
S1	3	4	4	4	3	2	1	5	CC3	I
S9	308	3	1	1	1	18	1	3	CC1	I

Sequence type: ST, *abcZ*: ATP-binding cassette transporter, *bglA*: beta-glucosidase, *cat*: catalase, *dapE*: succinyl-diaminopimelate desuccinylase, *dat*: D-amino acid aminotransferase, *ldh*: L-lactate dehydrogenase, *lhkA:* histidine kinase, CC: clonal complex.

Table S2. Comparative genomic analysis of virulence gene profiles in *Listeria monocytogenes* isolates.

Gene	Function	S1	S2– S8	S9
**Core Virulence Regulators**				
prfA	Master virulence regulator	+	+	+
sigB	General stress response sigma factor	+	+	+
virR (cesR)	Cell envelope stress regulator	+	+	+
agrA	Quorum-sensing system response regulator	+	+	+
codY	Global nutrient starvation regulator	+	+	+
**Pore-forming Toxins & Phospholipases**				
hly (plcC)	Listeriolysin O (LLO)	+	+	+
plcA	Phosphatidylinositol-specific phospholipase C	+	+	+
plcB	Broad-range phospholipase C	+	+	+
mpl	Zinc-metalloprotease (matures *PlcB*)	+	+	+
**Internalins**				
inlA	Invasion of epithelial cells	+	+	+
inlB	Invasion of hepatocytes and other cells	+	+	+
inlC	Intercellular spread and immune evasion	+	+	+
inlJ	Invasion and virulence in vivo	+	+	+
**Actin-Based Motility**				
actA	Actin nucleation and intracellular motility	+	+	–
**Cell Envelope Modification**				
oatA	Peptidoglycan O-acetylation	+	+	+
pgdA	Peptidoglycan N-deacetylation	+	+	+
mprF	Lysinylation of phosphatidylglycerol	+	+	+
dltA	Teichoic acid D-alanylation	+	+	+
gtcA	Teichoic acid glycosylation	+	–	–
Ami	Autolysin (virulence)	+	–	–
aut	Autolysin	+	–	–
chiA	Chitinase	+	+	+
**Stress Adaptation & Resistance**				
clpB, clpC,	ATP-dependent stress proteases	+	+	+

clpE, clpP				
htrA	Heat shock protease	+	+	+
bsh	Bile salt hydrolase	+	+	+
gadA	Glutamate decarboxylase (acid resistance)	+	–	–
gadB	Acid resistance	+	+	+
gad C	Glutamate/GABA antiporter (acid resistance)	+	+	+
fri	Ferritin-like protein	+	+	+
**Iron Acquisition**				
fur	Ferric uptake regulator	+	+	+
hupC	Heme uptake protein C	+	+	+
**Nutrient Acquisition**				
oppA	Oligopeptide transporter	+	+	+
eut_operon	Ethanolamine utilization	–	–	–
**Flagella & Motility**				
flaA	Flagellin	+	+	+
flgC/E	Flagellar biosynthesis	+	+	+
mogR	Transcriptional repressor of flagellar genes	+	+	+
**Biofilm & Adhesion**				
lapB	*Listeria* adhesion protein B	+	+	+
svpA	Persistence factor	+	+	+
degU	Biofilm regulation	+	+	+

Table S3. Core genome MLST analysis of *Listeria monocytogenes* isolates.

Sample	cgMLST profile ID	Allele Matches (out of 1748)	Percentage Similarity
S1	20537	1728	97.9%
S2	21857	1740	99.7%
S3	21857	1741	99.7%
S4	21857	1741	99.6%
S5	21857	1741	99.5%
S6	21857	1740	99.5%
S7	21857	1739	99.5%
S8	21857	1739	99.5%
S9	20879	1741	99.6%

Table S4. Metadata and accession numbers for isolates included in the phylogenetic tree.

Country of Isolation	Source	Accession Number
USA	clinical	SAMN02361175
USA	clinical	SAMN02383842
USA	clinical	SAMN02400146
USA	clinical	SAMN02400167
USA	clinical	SAMN02400173
USA	clinical	SAMN02400176
USA	clinical	SAMN02400179
USA	clinical	SAMN02400181
USA	clinical	SAMN02400185
USA	clinical	SAMN02400186
USA	clinical	SAMN02402780
USA	clinical	SAMN02402785
USA	clinical	SAMN02347439
USA	clinical	SAMN02351393
USA	clinical	SAMN02351394
USA	clinical	SAMN02351396
USA	clinical	SAMN02351399
USA	clinical	SAMN02351413
USA	clinical	SAMN02351419
USA	clinical	SAMN02358122
France	environmental/other	SAMEA4475960
France	environmental/other	SAMEA4475965
France	environmental/other	SAMEA4475966
Canada	environmental/other	SAMN12637253
United Kingdom	environmental/other	SAMN12659063
Canada	environmental/other	SAMN13113460
Canada	environmental/other	SAMN13113459
Canada	environmental/other	SAMN13113458
Canada	environmental/other	SAMN13113457
United Kingdom	environmental/other	SAMN13118741
United Kingdom	environmental/other	SAMN13118739
United Kingdom	environmental/other	SAMN13118747
United Kingdom	environmental/other	SAMN13118744
USA:IL	environmental/other	SAMN11268663
USA:NC	environmental/other	SAMN11268679
USA	environmental/other	SAMN11268684
USA	environmental/other	SAMN11268699
Switzerland	environmental/other	SAMN11268727
USA	environmental/other	SAMN11268725
USA:MD	environmental/other	SAMN11318834
USA:NY	environmental/other	SAMN12601274
USA:PA	environmental/other	SAMN12717644
USA:CA	environmental/other	SAMN13032359

USA:IL	environmental/other	SAMN08714154
USA:IL	environmental/other	SAMN08714152
USA:IL	environmental/other	SAMN08714153
USA:IL	environmental/other	SAMN08714156
USA:NC	environmental/other	SAMN08723992
USA:TX	environmental/other	SAMN09274462
USA:CA	environmental/other	SAMN09780225
USA:WI	environmental/other	SAMN10241725
USA:WI	environmental/other	SAMN10241726
USA:WI	environmental/other	SAMN10241723
USA:WI	environmental/other	SAMN10241724
USA:WI	environmental/other	SAMN10241728
USA:OH	environmental/other	SAMN10863107
USA:CA	environmental/other	SAMN11356658
USA:CA	environmental/other	SAMN11356657
USA:TX	environmental/other	SAMN11477210
USA:NY	environmental/other	SAMN12041563
USA:OH	environmental/other	SAMN11229974
USA:OH	environmental/other	SAMN11230094
USA:OH	environmental/other	SAMN11230744
USA:OH	environmental/other	SAMN11230745
USA:OH	environmental/other	SAMN11230891
USA:OH	environmental/other	SAMN11230857
USA:OH	environmental/other	SAMN11231014
USA:OH	environmental/other	SAMN11230890
USA:OH	environmental/other	SAMN11230888
USA:OH	environmental/other	SAMN11230892
USA:OH	environmental/other	SAMN11230853
USA:OH	environmental/other	SAMN11231015
USA:OH	environmental/other	SAMN11230836
USA:OH	environmental/other	SAMN11230902
USA:OH	environmental/other	SAMN11231023
USA:OH	environmental/other	SAMN11318968
USA:OH	environmental/other	SAMN11318948
USA:OH	environmental/other	SAMN11319102
USA:OH	environmental/other	SAMN11319114
USA:OH	environmental/other	SAMN11319524
Italy	environmental/other	SAMEA5546361
Italy	environmental/other	SAMEA5546362
Italy	environmental/other	SAMN04599878
Italy	environmental/other	SAMN04599954
Italy	environmental/other	SAMN04599955
Italy	environmental/other	SAMN04599959
USA:MN	environmental/other	SAMN04575430
USA:MI	environmental/other	SAMN04575442
USA:MI	environmental/other	SAMN04575443
Italy	environmental/other	SAMN04958319
USA:NY	environmental/other	SAMN06561803
USA:NY	environmental/other	SAMN06561798
USA:NY	environmental/other	SAMN06642530

USA:NY	environmental/other	SAMN06642533
USA:NY	environmental/other	SAMN06642532
USA:NY	environmental/other	SAMN06642531
USA:NY	environmental/other	SAMN06958588
USA:NY	environmental/other	SAMN06958592
USA:NY	environmental/other	SAMN06958589
Italy	environmental/other	SAMN04958320
USA:OH	environmental/other	SAMN12630136
USA:OH	environmental/other	SAMN12630137
USA:OH	environmental/other	SAMN12630138
USA:AL	environmental/other	SAMN12630141
USA:AL	environmental/other	SAMN12630143
USA:IL	environmental/other	SAMN12630153
USA:IL	environmental/other	SAMN12630156
USA:OH	environmental/other	SAMN12630124
USA:OH	environmental/other	SAMN12630125
USA:OH	environmental/other	SAMN12630129
USA:OH	environmental/other	SAMN12630128
USA:OH	environmental/other	SAMN12630127
USA:OH	environmental/other	SAMN12630126
USA:MO	environmental/other	SAMN12630123
USA:AR	environmental/other	SAMN12630145
USA:AL	environmental/other	SAMN12630140
USA:MO	environmental/other	SAMN12630122
USA:OH	environmental/other	SAMN12630139
USA:OH	environmental/other	SAMN12630131
USA:NC	environmental/other	SAMN12630121
USA:OH	environmental/other	SAMN12638092
USA:IA	environmental/other	SAMN12638103
USA:MI	environmental/other	SAMN12717643
USA:CO	environmental/other	SAMN12999235
USA:FL	environmental/other	SAMN13070472
USA:MO	environmental/other	SAMN13141258
USA:TX	environmental/other	SAMN11301412
USA:NE	environmental/other	SAMN11403764
USA:VA	environmental/other	SAMN11458020
USA:RI	environmental/other	SAMN11507390
USA:LA	environmental/other	SAMN11637125
USA:HI	environmental/other	SAMN11843916
USA:CA	environmental/other	SAMN11973966
USA:NC	environmental/other	SAMN12060627
USA:NJ	environmental/other	SAMN12142685
USA:LA	environmental/other	SAMN12340042
USA:TX	environmental/other	SAMN12358345
USA:TX	environmental/other	SAMN12384764
USA:SC	environmental/other	SAMN12476896
USA:MI	environmental/other	SAMN12523702
USA	environmental	SAMN14078538
USA	environmental	SAMN14078544

USA	environmental	SAMN14078535
USA	environmental	SAMN14078540
USA	environmental	SAMN14078542
USA	environmental	SAMN14078539
USA	environmental	SAMN14078547
USA	environmental	SAMN14078533
USA	environmental	SAMN14078534
USA	environmental	SAMN14078507
USA	environmental	SAMN14078548
USA	environmental	SAMN14078549
USA	environmental	SAMN14078541
USA	environmental	SAMN14078466
USA	environmental	SAMN14078474
USA	environmental	SAMN14078536
USA	environmental	SAMN14078546
USA	environmental	SAMN14078550
USA	environmental	SAMN14078532
USA	environmental	SAMN14078537
Drain 6b	SAMN15486404	SRR12179658
Raw product	SAMN14091610	SRR11077943
Drain 1a	SAMN15486400	SRR12179663
Drain 8b	SAMN14091708	SRR11078015
Raw product	SAMN14091734	SRR11077950
Drain 2b	SAMN15486402	SRR12179660
Drain 1b	SAMN15486405	SRR12179657
Drain 6b	SAMN15486409	SRR12179653
Raw product	SAMN15486410	SRR12179661
Drain 4b	SAMN14091736	SRR11077948
Drain 1b	SAMN14091733	SRR11077951
Drain 1b	SAMN15486408	SRR12179654
Drain 6b	SAMN15486407	SRR12179655
Drain 4b	SAMN15486403	SRR12179659
Drain 4b	SAMN14091735	SRR11077949
Drain 4b	SAMN15486406	SRR12179656
Raw product	SAMN15486401	SRR12179662
Drain 7b	SAMN14091732	SRR11077952
Drain 1b	SAMN14091725	SRR11077960
Drain 8b	SAMN14091729	SRR11077956
